# Serrated Polyps and Their Alternative Pathway to the Colorectal Cancer: A Systematic Review

**DOI:** 10.1155/2015/573814

**Published:** 2015-04-07

**Authors:** Łukasz Szylberg, Marlena Janiczek, Aneta Popiel, Andrzej Marszałek

**Affiliations:** ^1^Department of Clinical Pathomorphology, Collegium Medicum in Bydgoszcz, Nicolaus Copernicus University in Torun, Sklodowskiej-Curie 9, 85-094 Bydgoszcz, Poland; ^2^Department of Oncologic Pathology, Poznan University of Medical Sciences, Poznan, Poland

## Abstract

Colorectal cancer (CRC) is the third most frequently diagnosed cancer in the world. For a long time, only one pathway of colorectal carcinogenesis was known. In recent years, a new “alternative” pathway through serrated adenoma was described. Recent meta-analysis estimated these cancers as about 10% to 30% of all CRCs. Serrated polyps are the second most popular groups of polyps (after conventional adenomas) found during colonoscopy. Serrated polyps of the colon are clinically and molecularly diverse changes that have common feature as crypt luminal morphology characterized by glandular serration. Evidence suggests that subtypes of serrated polyps, particularly TSA and SSA/P, can lead to adenocarcinoma through the serrated pathway. Moreover, the data indicate that the SSA/P are the precursors of colorectal carcinoma by MSI and may be subject to rapid progression to malignancy. An important step to reduce the incidence of CRC initiated by the serrated pathway is to improve the detection of serrated polyps and to ensure their complete removal during endoscopy. Understanding of the so-called serrated carcinogenesis pathway is an important step forward in expanding possibilities in the prevention of CRC.

## 1. Introduction

Colorectal cancer (CRC) is the third most frequently diagnosed cancer in the world. Each year about 600,000 people die from it [1]. Over 90% of CRCs are adenocarcinomas [2]. For a long time, only one pathway of colorectal carcinogenesis was known. Vogelstein et al. described it as “classical” pathway through adenoma-adenocarcinoma sequence [3]. In recent years, a new “alternative” pathway through serrated adenoma was described. Adenocarcinomas developed from serrated lesions were first described by Jass and Smith [4]. They estimated these cancers as about 10% to 30% of all CRCs. Serrated polyps are the second most popular groups of polyps (after conventional adenomas) found during colonoscopy [5]. In the literature two terms are used and accepted for serrated lesions such as serrated polyps or serrated adenomas [6]. According to the 2010 WHO classification, serrated polyps are divided into three subgroups such as sessile serrated adenoma/polyp (SSA/P), traditional serrated adenoma (TSA), and hyperplastic polyps (HPs) [7]. HP has been recognized as a distinct type of these lesions in the colon. In recent years, it has been challenged that HP was considered as benign lesion, but numerous studies have shown the malignant nature of this lesion. In this regard, nature of HP is clinically important. The appearance of this type of polyp in the colon should lead to oncological alert and obligatory procedure of polyp removal [8]. Serrated pathway leading to the development of colon cancer is not homogeneous. It depends on the genetic and molecular variation [9]. Understanding carcinogenesis pathways can improve the treatment process and help prevent the CRC development.

This review presents the current knowledge of serrated polyps and their pathways leading to CRC. Serrated polyps are interdisciplinary problem among gastroenterologists, pathologists, and oncologists.

## 2. Classification of Serrated Polyps

### 2.1. General Classification

Serrated adenomas were first described in 1990 by Longacre and Fenoglio-Preiser [10]. Serrated polyps are heterogeneous lesions. Histologically they are characterized by glandular serration. Colonic epithelial crypts show luminal “saw-toothed” pattern. This feature is considered as a result of cell growth in combination with the rotation of the delayed migration or failure of cells apoptosis which lead to accumulation of the epithelial cells. It is now recognized that several different subtypes of serrated polyps exist and can lead to a subset of invasive cancer by serrated pathway [11]. Serrated polyp nomenclature is in evolution. The latest classification of the World Health Organization (WHO) classifies them into three main groups: HP, SSA/P, and TSA [12].

#### 2.1.1. Hyperplastic Polyps

HP represents more than three-quarters of serrated polyps [13]. HPs are flat or sessile, pale lesions, generally do not exceed 5 mm, and are usually located at the ends of the folds of the rectal mucosa. In colorectum, HPs are often larger and more difficult to visualize in endoscopic procedure. HPs develop at a younger age than conventional adenomas, but their frequency does not seem to significantly increase after 50 years of age [14].

#### 2.1.2. Sessile Serrated Adenomas/Polyps

SSA were first identified in 2003 by Torlakovic et al. [15]. They Represent 15 to 20% of all serrated polyps [16]. SSA are flat or slightly elevated. These lesions occur most commonly in the proximal colon and are typically measuring more than 5 mm. Histologically, SSA differ from HP, the presence of abnormal architecture characteristics secondary to abnormal proliferation. In HP, the proliferation zone is located at the bottom of the crypts. However, in SSA, crypts proliferation leads to an increase in the crypts being asymmetric of T-shaped or inverted L-shaped structures. Other characteristic features include the presence of mature goblet cells at the base of the crypts, hyperserration throughout the base or in the crypts, and muscle pseudoinvasion. Dysplasia is absent [6, 17, 18].

#### 2.1.3. Traditional Serrated Adenomas

TSAs are relatively not very frequent polyps estimated up to 5% serrated polyps in Western countries with higher prevalence in Asia, especially in Korea [19]. Compared to the SSA, TSAs are found on the left side of the colon and in the elderly. The architecture of TSA is often more complicated than villous or tubulovillous adenoma, but with visible serration. Ectopic crypt foci are present in the TSA, specified by presence of crypts at their base (not sitting at the level of muscularis propria). This morphological feature is useful to distinguish them from the SSA. Neoplastic cells are characterized by abundant eosinophilic cytoplasm and elongated, pencillate nuclei. Dysplasia of TSA is usually clinically “benign” comparing appearance of dysplasia associated with conventional adenoma and low proliferation properties [20].

## 3. Epigenetic and Genetic Aspects of Serrated Polyps

**Figure 1:**
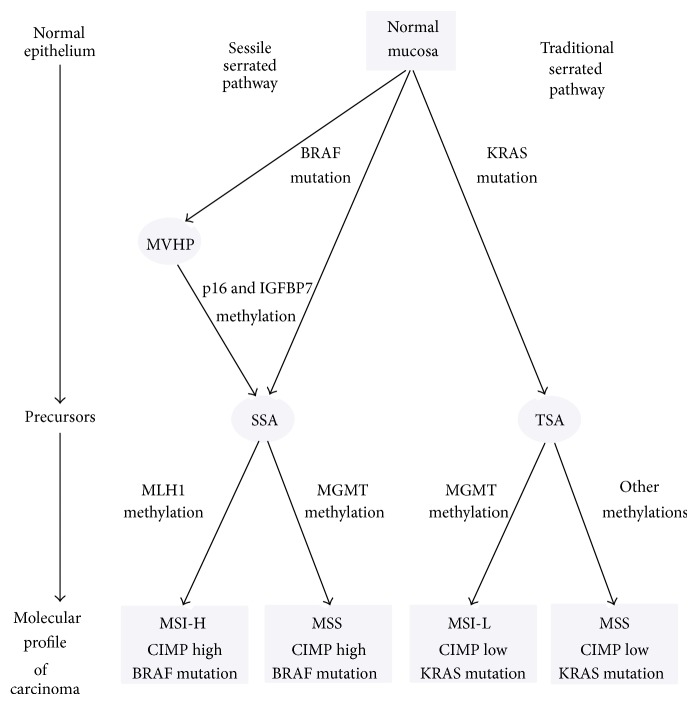
Sessile serrated and traditional serrated pathways. The first pathway consists of molecular profile cancers which are CpG island methylator phenotype-high (CIMP-high) and BRAF mutation positive with microsatellite instability being high (MSI-H) or stability (MMS). The precursor of these cancers may be MVHP and SSA. The second pathway consists of CpG island methylator phenotype-low (CIMP-low), KRAS mutations positive with microsatellite stability or instability being low (MSI-L). The precursor lesion in this second pathway may be TSA. MVHP: microvesicular hyperplastic polyp; SSA: sessile serrated adenoma; TSA: traditional serrated adenoma.

Classical “adenoma-carcinoma” sequence which includes changes from the normal mucosa towards the carcinoma belongs to specific and well defined genetic alterations such as APC (adenomatous polyposis coli), and oncogenes as KRAS (Kristen rat sarcoma viral oncogene homolog), DCC (deleted in CRC), and TP53 (tumor protein 53) [21]. Development of colon cancer is caused by a cascade of genetic mutations which leads to a progressive disordered DNA replication and accelerated colonocyte replication. Progressive cascade of genetic mutations causes the transformation from normal mucosa through benign adenomas to adenomas with high grade dysplasia and finally into invasive adenocarcinoma [18, 22]. These four stages of CRC carcinogenesis are characterized by excessive activation of oncogenes and inactivation of tumor suppressor genes. In the first phase, inactivation of the APC gene causes the development of adenomas. In the second phase, KRAS mutations promote adenoma growth. In the third phase, the LOH (loss of heterozygosity) supports the progression of adenoma. In the last step, inactivation of TP53 triggers the final transition to the cancer. This sequence is present in 60% of CRC cases [18]. It was considered that about 20% of CRCs develop on the base of serrated lesions through another pathway called “serrated pathway.” It is associated with the sequence of genetic and epigenetic alternations [23]. According to Torlakovic et al. [15], there are two main types of genetic sequences with their subtypes which lead to SAC (serrated adenocarcinoma) (Figure 1). Before 2003, serrated adenoma which includes TSA and SSA was named as hyperplastic polyps. SSA revealed BRAF mutation, high level of CpG island methylation (CIMP-high), and MLH1 gene hypermethylation [24, 25]. Non-CIMP was attributed to two groups. The first one is closely associated with presence of TP53 mutations and location of the cancer in distal colon. The second one is characterized by low periodicity of hypermethylation and gene mutation which is specified for the cancer located usually in rectum [26]. Methylation of CpG islands is a proper way of reducing gene expression (more methylation means less expression). If the silenced gene is tumor suppressor gene, then loss of function simplifies carcinogenesis. Epigenetics describes the study of dynamic alterations in the transcriptional potential of a cell but does not engage in DNA sequence. Contemporary role of epigenetic events leads to development of diagnostic tests. It can be used in prediction of biological aggressiveness, proper diagnosis, and clinical response of certain cancers [27]. Identification of DNA methylated markers by methylation-specific PCR (MSP) or non-MSP prescribed how PCR primer should be designed. Epigenetic alterations often rather than genetic changes could be used as early diagnostic tools. There are useful early methylated genes such as SLC5A8, MINT1, MINT31, SFRP1, SFRP2, CDH13, CRBP1, RUNX3, p14ARF, HLTF, ITGA4, and CDKN2A. The hypermethylation promoter can be detected from tissue biopsies, blood samples, stool, peritoneal fluid, and urine [28]. Moreover, epigenetic alterations may influence drug resistance. An interesting example is 5-fluorouracil (FU), which is conventional chemotherapeutic agent used for CRC treatment. 5-FU works by initiation apoptosis through several apoptosis regulatory genes. If targeted genes are silenced by DNA methylation in CRC cells, the lower response to 5-FU therapy was observed [29]. Silencing gene by methylation is an epigenetic event [30]. CIMP-high is clinically associated with older age, female sex, and proximal tumor location [31]. According to presence of MLH1-DNA mismatch repair gene is associated with MSI-H (microsatellite instability-high) and good prognosis. On the other hand, different type of methylation can lead to MSS (microsatellite stability) and has very poor prognosis [32]. TSAs are usually related to low grade dysplasia [14]. TSA is frequently associated with KRAS mutation but BRAF mutation can also occur. They are early molecular alterations in serrated lesions [33]. Additional feature of serrated pathway is silencing of the DNA repair gene O-6-methylguanine-DNA methyltransferase (MGMT) which is associated with CIMP-low. MGMT promoter methylation and silencing have been associated with guanine more than adenine mutations and MSI-low [34]. Based on Stefanius et al., BRAF mutation is more frequent than KRAS in all serrated adenocarcinomas. BRAF mutation is more specific to serrated adenocarcinomas than KRAS. KRAS mutation is linked with CIMP-low but is independent of MSI status [26]. BRAF or KRAS mutations may result in mitogen activated protein kinase (MAPK) activation. BRAF and KRAS mutation are among to the intracellular MAPK cascades responsible for cell growth [35]. However, many studies claimed that BRAF mutation is characteristic to the serrated pathway [25, 36]. Colon cancers, which occurred after preventive colonoscopy, may represent cases such as missed cancers and development of cancers from missed or incompletely removed adenomatous polyps or rapid malignant progression of serrated polyps. Missed cancers have shown MSI and CIMP which they are both signature of serrated pathway. Serrated pathway of carcinogenesis could be related to duration of interval [37].

## 4. Molecular Pathological Epidemiology of Serrated Lesions

Molecular pathological epidemiology (MPE) is an integrative molecular and population health science, which is related to the molecular pathogenesis and variety of disease processes [38]. MPE provided contrast data to conventional epidemiological research which include genome-wide association studies (GWAS) [39]. The term “molecular pathological epidemiology” was used by Ogino and Stampfer in 2010 [40]. This term has been widely accepted by the scientists and clinicians [41, 42]. MPE colorectal precancerous changes can provide unique opportunity to study effects of diet, lifestyle, and environmental factors on individual pathways of carcinogenesis. MPE lesions and intestines can identify causative exposures associated with the initiation and progression of cancer. It may help us to better understand CRC development and facilitate personalized prevention, screening, and treatment [38].

Recent research revealed the relation between the molecular pathology of CRC and exposure to risk factors. Samowitz and Limusi et al. demonstrated the relationship between CIMP-high and BRAF-mutated CRC and cigarette smoking [43, 44]. Nishihara et al. showed that lower risk of CIMP-high cancer is connected with former smokers with long term cessation [45]. Another study conducted by the Burnett-Hartman and colleagues focused on the relationship between the molecular pathology of serrated lesions and exposure to smoking cigarette. It has demonstrated statistical significance between CIMP-high and BRAF mutation and serrated lesions. They also observed the relationship between CIMP-low/negative and BRAF-wild-type serrated lesions and cigarette smoking. Therefore, the authors suggest that smoking may affect the initiation of serrated lesions, but not depending on BRAF mutation and CIMP. Another factor demonstrating the relationship between CIMP-high and BRAF mutations serrated lesions was high BMI [46]. However, in this case, no significant correlation with CRC was found [47, 48]. Ethnicity and genetic variation are also evaluated in relation to molecular subsets of serrated lesions. The study showed that there is a positive association between CIMP-high in serrated lesions and Caucasians. Such correlation could potentially explain epidemiologic differences occurring between ethnic and racial groups [46]. However, in the study of colon cancer a clear relation to this factor has not been established [49, 50].

## 5. Serrated Polyposis Syndrome

Serrated polyposis syndrome (SPS) is now also known as hyperplastic polyposis syndrome described in the early seventies. This condition predisposes to cancer of the colon with the risk of 25–40% [51]. Although the first patients with SPS were documented in 1970, it has been recognized as the condition with genetic potential and precursor of CRC recently [52]. SPS are more common in people of Northern Europe and have relationships with cigarette smoking [53]. In addition to many serrated polyps, adenomas of the colon may be a part of this syndrome as they are identified in 85% of SPS patients [54]. SPS remains the least known and least understood changes in the colon. For a long time this syndrome was considered as having no clinical consequence, based on the opinion that serrated polyps are benign [55]. For decades it was thought that the malignant transformation of conventional adenomas is only a single mechanism underlying the development of CRC [56]. In the late nineties of the XX century, a number of important observations moving major paradigm shift in the way of initiation and progression of CRC [57]. These observations suggest that some serrated polyps may also act as precursor lesions in CRC development through alternative carcinogenesis pathways existing alongside traditional adenoma sequence. Observations suggest that a subset of serrated polyps can develop functions associated with cancer. The first clinical criteria for the recognition of serrated polyposis were created in 2000 [58]. This condition is determined to extract it from the familial adenomatous polyposis (FAP) and it became necessary to introduce appropriate criteria for observation of serrated polyps that are often in the distal part of the colon and rectum which did not include the definition of FAP [59]. The lesions in serrated polyposis do not exhibit the characteristics that distinguish them from sporadic serrated polyps, except that they are extremely numerous. The large polyps are often in the colon; small polyps are often seated distributed throughout the colon and rectum. The current revised criteria published in 2010 define serrated polyposis which includes any one of the following criteria: (a) at least five serrated polyps in the proximal sigmoid colon and at least two greater than 10 mm, (b) any number of serrated polyps proximal to the sigmoid colon in first degree relative of patients with serrated polyposis, or (c) more than 20 serrated polyps of any size, but distributed throughout the colon [55]. However, the criteria for serrated polyposis differ in publications as some authors indicate large phenotypic changes and coincidence with sporadic polyps. Serrated polyposis is a relatively common disease and in such patients history of family CRC is relatively frequent phenomenon occurring up to 60% [60].

The serrated polyposis progression may be associated with premature aging of normal mucosa (e.g., the widespread gene promoter hypermethylation). Despite the large number of serrated polyps in the large intestine, only one-third has BRAF V600E mutation, a molecular feature of a serrated pathway. It suggests that the serrated lesions could develop from nonhomogeneous pathway [36, 61].

## 6. Clinical Challenge Associated with Serrated Pathway

Detailed knowledge of serrated pathway leading to CRC has important clinical implications for the detection, monitoring, and treatment [62]. Colorectal serrated polyps differ in clinical and molecular aspects which is reflected in different carcinogenesis pathway [63]. Unfortunately, the clinical data is limited, as it has been only a few years serrated that polyps are treated as potentially malignant lesions [64]. There are still many unanswered questions about the transition of serrated polyps into invasive cancer which differs from the classical adenoma-carcinoma sequence and whether its transformation is progressing faster or slower. Recent limited studies indicate that developing CRCs take place faster [65] and therefore it is important to remove these lesions during colonoscopy to prevent the development of invasive cancer. Cancer formed on the base of serrated lesion is mostly located in the proximal part of the large intestine. Serrated lesions have a distinct endoscopic appearance, but more difficult to diagnose than conventional polyps. Endoscopist should be trained in the diagnosis of serrated polyps to increase the level and effectiveness of colonoscopy. According to the recommendation of Rex et al. all serrated lesions proximal to the sigmoid colon and all serrated lesions in the rectosigmoid more than 5 mm in size should be completely removed. In addition, recommendations for postpolypectomy surveillance include monitoring patients with serrated lesions in colon and their families [17].

## 7. Conclusion

Understanding of the so-called serrated carcinogenesis pathway is an important step forward in expanding possibilities in the prevention of CRC. Serrated polyps of the colon are clinically and molecularly diverse changes that have common feature as crypt luminal morphology characterized by glandular serration. Evidence suggests that subtypes of serrated polyps, particularly TSA and SSA/P, can lead to adenocarcinoma through the serrated pathway. Moreover, the data indicate that the SSA/P are the precursors of colorectal carcinoma by MSI and may be subject to rapid progression to malignancy. SSA/P and MSI-H colorectal carcinomas are more common in the proximal colon. An important step to reduce the incidence of CRC initiated by the serrated pathway is to improve the detection of serrated polyps and to ensure their complete removal during endoscopy.
